# “Mirror, mirror, on the wall, without you, I will fall”: investigation into body dysmorphic disorder from an attachment perspective

**DOI:** 10.3389/fpsyt.2026.1846717

**Published:** 2026-07-03

**Authors:** Liang Zhang, Xinze Liu, Yichao Lv, Xinyuan Zou, Yanqiang Tao, Xiangping Liu, Shujian Wang

**Affiliations:** 1 Northeast Agricultural University, Harbin, China; 2 Beijing Key Laboratory of Applied Experimental Psychology, National Demonstration Center for Experimental Psychology Education, Faculty of Psychology, Beijing Normal University, Beijing, China; 3 East China Normal University, Shanghai, China; 4 Department of Psychology, Beijing Normal University, Beijing, China

**Keywords:** appearance-based rejection sensitivity, attachment anxiety, body dysmorphic disorder, cross-sectional study, gender differences

## Abstract

**Objective:**

Body dysmorphic disorder (BDD) is a prevalent concern among young adults. However, the underlying mechanisms of BDD development remain elusive. This study aims to investigate the intricate relationship between attachment styles and BDD symptoms, with appearance-based rejection sensitivity (ARS) as a mediating factor and gender as a moderator.

**Methods:**

A total of 815 young adults participated, completing a battery of questionnaires including the Revised Adult Attachment Scale (RAAS), Appearance-Based Rejection Sensitivity Scale (ARSS), and Scale of Body Image (SBI).

**Results:**

Data indicated a positive association between attachment anxiety and BDD symptoms, with ARS found to mediate this link. Furthermore, gender differences were observed to moderate the relationship between ARS and BDD symptoms.

**Conclusion:**

This study sheds light on the foundational mechanisms of BDD, tracing its origins to early caregiver-infant bonds and highlighting the enduring impact of ambivalent care on body image perceptions. Additionally, the identification of ARS as a specific contributing factor to BDD onset underscores its significance in understanding and addressing this disorder. By considering the influence of social norms and cultural context, gender differences in the association between ARS and BDD symptoms are elucidated.

## 1 Introduction

According to the DSM-5-TR, body dysmorphic disorder (BDD) is characterized by persistent preoccupation with perceived defects or flaws in physical appearance that are not observable or appear slight to others (1). Individuals with BDD symptoms often engage in repetitive behaviors, such as frequent mirror checking, excessive weightlifting, or making comparisons with others regarding their shape or appearance (2). Notably, BDD affects a significant portion of adolescents, with prevalence rates ranging from 2% to 5% (3). Despite its high prevalence in this age group, the underlying mechanisms contributing to the development and maintenance of BDD remain poorly understood (4).

### 1.1 Attachment anxiety and BDD symptoms

Attachment theory provides a developmental framework for understanding how early caregiver-child relationships shape later interpersonal expectations, affect regulation, and vulnerability to psychopathology. Originally formulated by Bowlby, the theory proposes that human beings possess an attachment behavioral system that motivates proximity seeking toward significant others under conditions of threat, distress, or uncertainty (5). When caregivers are consistently available and responsive, they serve as a secure base and safe haven, enabling children to regulate distress and develop relatively positive models of the self and others (5, 6). By contrast, inconsistent or insensitive caregiving may undermine felt security and increase vulnerability to anxiety, concerns about rejection, and maladaptive emotion-regulation strategies (7, 8). Ainsworth et al. later extended and empirically operationalized Bowlby’s theory through the Strange Situation procedure, identifying secure, anxious, and avoidant attachment patterns (9). For the present study, the anxious pattern is particularly relevant because it provides a developmental basis for adult attachment anxiety.

Although early attachment patterns do not determine adult attachment in a fixed or irreversible manner, attachment theory assumes that repeated caregiver-child interactions are gradually internalized into relatively stable internal working models of the self and others (5, 10). Longitudinal and meta-analytic evidence indicates moderate continuity in attachment from infancy and childhood into later development, while also showing that attachment representations may be revised through subsequent relational experiences (11, 12). During adolescence and adulthood, these internal working models are increasingly expressed in peer, romantic, and other close relationships, making adult attachment anxiety a developmentally continuous but modifiable extension of earlier anxious attachment experiences (13, 14).

Attachment anxiety is characterized by a strong desire for closeness, doubts about one’s worthiness of care, uncertainty about others’ availability, and heightened fear of abandonment (6). Individuals high in attachment anxiety tend to intensify proximity seeking and remain highly vigilant to signs that important others may be unavailable, rejecting, or dissatisfied (7, 8). This pattern can be understood in terms of both the cognitive and affective consequences of insecure internal working models. Cognitively, individuals high in attachment anxiety tend to hold a negative model of the self and an uncertain model of others: they often doubt their own worthiness of care while questioning whether important others will remain available and responsive (10, 13). These working models guide attention, interpretation, and memory in close relationships. As a result, ambiguous interpersonal cues may be interpreted as signs of rejection, disapproval, or abandonment, and individuals may become preoccupied with monitoring whether they are valued by others (6). In this sense, attachment anxiety is not merely a general desire for closeness, but also a cognitive schema through which the self is evaluated as vulnerable to rejection and others are perceived as unreliable sources of reassurance. Affectively, attachment anxiety is associated with hyperactivation of the attachment system. When relational threat is perceived, individuals high in attachment anxiety tend to experience intensified distress, fear of abandonment, anger, sadness, and difficulty down-regulating negative emotions (7, 8). Because reassurance from others is experienced as uncertain or temporary, these individuals may repeatedly seek confirmation of acceptance while remaining vigilant to further signs of rejection. Thus, adult attachment anxiety involves a cognitive-affective pattern in which negative self-representations and uncertain expectations of others are coupled with heightened emotional reactivity and persistent reassurance seeking.

This attachment framework is useful for understanding why attachment anxiety may contribute to BDD symptoms. Although BDD symptoms center on perceived defects in appearance, such concerns often arise within a social-evaluative context and are maintained by cognitive and emotional processing biases related to appearance threat (15). For individuals high in attachment anxiety, appearance may become a salient cue through which relational value is inferred. A perceived physical flaw may therefore be experienced not only as an aesthetic problem, but also as evidence that one is less acceptable, less lovable, or more likely to be rejected by others. This interpretation is consistent with recent work extending cognitive-behavioral models of BDD by showing that attachment anxiety and self-ambivalence are relevant to BDD symptoms (16).

Accordingly, individuals with attachment anxiety may monitor their appearance as a way to manage relational insecurity. Repetitive mirror checking, comparison with others, concealment, excessive grooming, and attempts to improve appearance can be understood as behavioral efforts to reduce distress and prevent rejection. However, these strategies may paradoxically maintain or intensify BDD symptoms by increasing attention to perceived flaws and reinforcing the belief that social acceptance depends on appearance (15, 17). Therefore, attachment anxiety may increase BDD symptoms through a cognitive-affective pathway: insecure internal working models foster negative self-evaluations, hyperactivating strategies amplify distress and rejection vigilance, and appearance-related concerns become a specific domain in which these relational fears are expressed.

H1: Attachment anxiety positively predicts BDD symptoms.

### 1.2 The mediating role of appearance-based rejection sensitivity

In investigating the mechanism linking attachment anxiety and BDD symptoms, one crucial construct to consider is appearance-based rejection sensitivity (ARS). ARS is a specific aspect of rejection sensitivity—a personality trait characterized by adolescents perceiving a high risk of rejection in relationships due to their appearance, shape, or figure (18).

From a social interaction perspective, adolescents with attachment anxiety may experience frequent rejection and abandonment due to inconsistent caregiving experiences during childhood (5, 19). Paradoxically, they may also possess a strong desire for intimacy and closeness (20). Consequently, these individuals may be hypersensitive to cues of rejection (21).

From a cognitive standpoint, rejection is perceived as a negative stimulus, prompting adolescents with attachment anxiety to develop heightened vigilance towards potential threats in relationships (22). This vigilance may lead them to interpret neutral or vague hints as potential threats of rejection.

Previous research has explored the relationship between attachment anxiety and rejection sensitivity (23, 24). However, in the context of investigating BDD symptoms, it is essential to consider ARS as a specific dimension of rejection sensitivity that may uniquely contribute to BDD. In social interactions, adolescents’ concerns about negative evaluations of their appearance can lead to body image dissatisfaction, acceptance of cosmetic surgery, or dysfunctional investment in appearance (25). Additionally, ARS serves as a cognitive factor in the onset of BDD, potentially acting as a stimulus or reinforcement for subsequent BDD symptoms (17). Adolescents who place significant value on their appearance may become overly attentive to their physical attributes, exacerbating their vulnerability to BDD (26).

Despite the importance of ARS in understanding the development of BDD, existing research has predominantly focused on other sources of rejection, such as maternal rejection, parental image concerns, or peer teasing (27, 28). However, attachment anxiety, which reflects the quality of early bonds between infants and parents, has profound implications for adolescents’ mental health (29). Investigating attachment anxiety can shed light on the origins of rejection in relationships and its association with ARS.

Moreover, while scholars have often emphasized behavioral manifestations of BDD, such as cosmetic surgery or body image concerns, exploring the role of ARS in the context of BDD can provide a deeper understanding of body image issues among adolescents. Therefore, the current study aims to bridge the gap between attachment anxiety, ARS, and BDD, shedding light on the underlying mechanisms of BDD as a complex psychological construct.

H2: ARS serves as the mediator in the relationship between attachment anxiety and BDD symptoms.

### 1.3 Gender as a moderator

In addition to investigating the mechanism of BDD from the perspective of attachment anxiety, the current study aims to examine the role of gender as a moderator in the relationship between ARS and BDD, taking into account the social norms prevalent in China. These norms suggest that males’ social status is often associated with their success, while females’ value is frequently linked to their body shape (30). Consequently, it is plausible that females may be more vulnerable to BDD due to the emphasis placed on their physical appearance.

However, previous research on the role of gender in BDD has yielded mixed findings. This inconsistency may be attributed to researchers focusing on different racial or ethnic groups, resulting in gender roles becoming confounded by other demographic factors (31, 32). Additionally, variations in the facets of BDD assessed and the conceptual frameworks utilized to evaluate gender differences further contribute to this complexity (33–35).

To address this issue, the current study aims to comprehensively examine gender issues in BDD, providing practitioners with a clearer understanding for effective treatment strategies. Furthermore, from a social perspective, females are often evaluated based on their weight, shape, or appearance, whereas these criteria may not hold the same significance for males. Therefore, we propose the following hypothesis:

H3: Gender moderates the association between ARS and BDD. The mediating effect of ARS was more pronounced for females compared to males.

**Figure 1:**
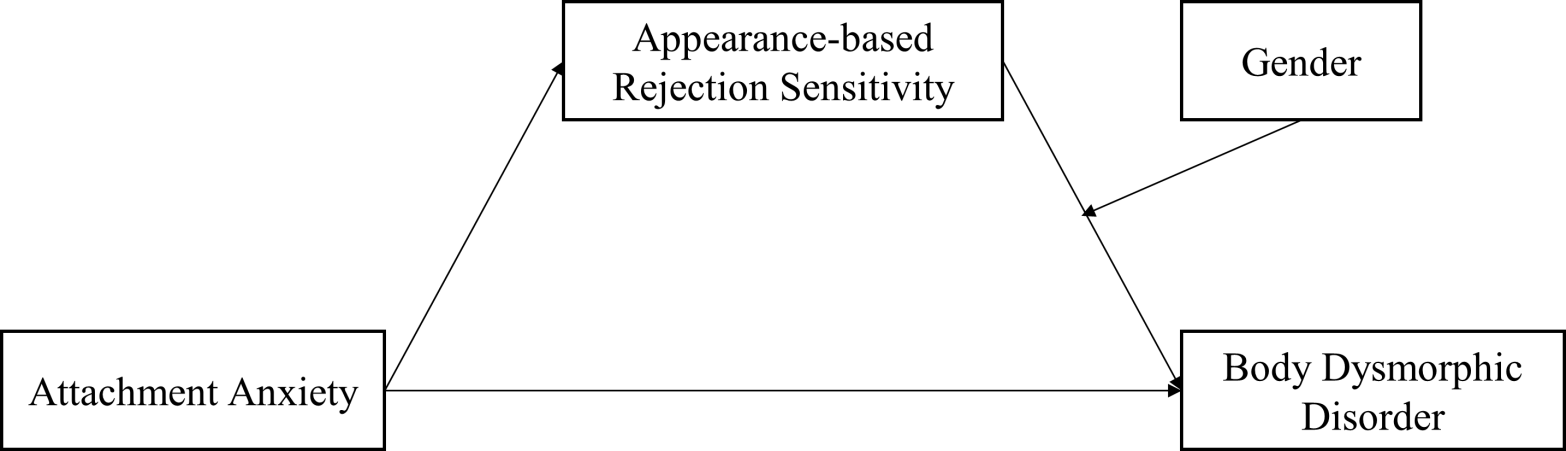
The proposed moderated mediation model.

In summary, the present study proposed a moderated mediation model as shown in **Figure 1**.

## 2 Methods

### 2.1 Participants and procedure

Data were collected through an online questionnaire administered at Northeast Agricultural University. University staff members distributed the questionnaire link to student chat groups, inviting students to participate. A total of 888 responses were obtained. Following screening procedures, 815 valid questionnaires were retained for analysis. Participants’ ages ranged from 16 to 25 years (*M* = 18.79, *SD* = 1.08), with detailed demographic information presented in **Table 1**. Electronic informed consent was obtained from all participants, and the ethics committee of Beijing Normal University approved the current research (IRB number: 202409290170).

**Table 1:** Demographic characteristic (N = 815).

Variables	N	%
Gender		
Female	318	39.0%
Male	497	61.0%
Only Child		
Yes	443	54.4%
No	372	45.6%
Romantic relationship		
Yes	619	76.0%
No	196	24.0%
Subjective economic status		
Very low	94	11.5%
Low	222	27.2%
Medium	462	56.7%
High	34	4.2%
Very high	3	0.4%
Father education level		
Junior high and below	373	45.8%
Senior high	225	27.6%
Bachelor	167	20.5%
Master	21	2.6%
Doctor	7	0.9%
Reluctant to disclose	22	2.7%
Mother education level		
Junior high and below	463	56.8%
Senior high	173	21.2%
Bachelor	148	18.2%
Master	5	0.6%
Doctor	1	0.1%
Reluctant to disclose	25	3.1%

### 2.2 Measures

#### 2.2.1 Demographic information

Relevant demographic data were collected via self-report, encompassing age, gender, subjective economic status, parental education level, relationship status, and sibling status.

#### 2.2.2 Revised Adult Attachment Scale

Attachment anxiety was assessed using the anxiety subscale of the Revised Adult Attachment Scale (RAAS), developed by Collins (36) on the basis of earlier adult attachment measurement work by Collins and Read (13) and adapted into Chinese by Wu et al. (37). The RAAS is an 18-item self-report measure designed to assess individual differences in adult attachment across three dimensions: close, depend, and anxiety. The anxiety subscale captures concerns about rejection, abandonment, and others’ availability in close relationships. Previous research has used the RAAS to examine adult attachment representations, internal working models, and relationship quality. Validation studies have demonstrated satisfactory reliability and validity for the RAAS in China and in other cultural settings (37, 38). In the present study, only the anxiety subscale was used because the theoretical focus was attachment anxiety, with higher scores indicating greater attachment anxiety. The anxiety subscale demonstrated acceptable reliability in this study (Cronbach’s α = 0.79).

#### 2.2.3 Appearance-Based Rejection Sensitivity Scale

Appearance-based rejection sensitivity was measured using the Appearance-Based Rejection Sensitivity Scale (ARSS), developed by Park (18) and adapted into Chinese by Deng et al. (39). Park conceptualized appearance-based rejection sensitivity as a personality-processing system characterized by anxious expectations of rejection based on one’s physical appearance (18). The ARSS contains 15 appearance-related social scenarios. For each scenario, participants rate their anxiety about possible rejection and their expectation that rejection would be based on appearance. The two ratings are multiplied for each scenario, and the mean score across the 15 scenarios is calculated, with higher scores indicating greater appearance-based rejection sensitivity. The ARSS has been used in previous studies to examine appearance threat, self-esteem, affect, belongingness, body dysmorphic symptoms, and cosmetic surgery acceptance (18, 25, 40). It has also been applied in studies of body dysmorphic disorder and body dysmorphic concerns in both clinical and adolescent samples (17, 27, 28, 41). The Chinese version has shown satisfactory reliability and validity among Chinese college students (39). In this study, the ARSS showed strong internal consistency (Cronbach’s α = 0.91).

#### 2.2.4 Scale of Body Image

BDD symptoms were assessed using the Scale of Body Image (SBI), developed by Lu et al. (42). The SBI is a 21-item self-report scale developed in the Chinese context to assess subjective body-image disturbance and distress related to perceived changes or defects in one’s body image. Items are rated on a 4-point scale ranging from 1 = none to 4 = almost always, with higher scores indicating more severe body-image-related distress. SBI has been widely used in Chinese research to assess body-image disturbance in appearance-relevant populations, including women seeking weight loss, patients with vitiligo, and individuals seeking facial cosmetic surgery (43, 44). Previous studies have applied the scale to examine associations between body-image disturbance and self-esteem, personality, social support, social appearance anxiety, and fear of negative evaluation. These studies suggest that the SBI is suitable for assessing body-image-related distress in Chinese samples, particularly in contexts where appearance concerns are psychologically salient (43, 44). Because the present study used a nonclinical college student sample, the SBI was used as a dimensional self-report indicator of BDD-related symptoms rather than as a diagnostic instrument for BDD. In this study, the SBI demonstrated strong internal consistency (Cronbach’s α = 0.93).

### 2.3 Data analysis

All data analysis were performed in R version 4.2.2. First, Harman’s single-factor test was used to test for common method bias (45). The factor analysis extracted 10 factors (eigenvalue > 1), with the most important factor accounting for 23.40% (less than 40%) of the variance, suggesting no significant common method variance. Second, a multicollinearity analysis was conducted to check whether the predictor variables were related to each other. The variance inflation factor (VIF) of all predictors were 1.02~2.92 (less than 5), reflecting no severe multicollinearity. Next, the kurtosis and skewness values were used to test whether variables had a normal distribution. Hair et al. (46) and Byrne (47) argued that data is considered to be normal if skewness is between -2 to +2 and kurtosis is between -7 to +7. According to this criterion, the variables were in line with normal distribution. The R package lavaan (48) was used to examine the mediating effect and moderated mediating effect. According to Muller’s (49) recommendation, all predictors were centered prior to analysis.

## 3 Results

### 3.1 Preliminary analyses

An independent sample *t*-test was performed to explore the gender differences in main variables. **Table 2** presents the descriptive information and *t*-test results of AA, ARS, and BDD. The *t*-test results showed that female participants exhibited significantly higher ARS than males (*t* = 3.20, *p* < 0.01); however, the effect size of this gender difference was small (Cohen’s *d* = 0.23). Thus, this difference should be interpreted as statistically reliable but modest in practical size.

**Table 2:** Descriptive statistics and t-test results.

Variable	M	SD	Skew	Kurtosis	Female M ± SD	Male M ± SD	t	p	Cohen’s d
AA	2.78	0.77	0.16	-0.19	2.81 ± 0.78	2.73 ± 0.76	1.49	0.14	0.11
ARS	8.40	5.15	1.12	1.69	8.86 ± 5.22	7.70 ± 4.96	3.20	<0.01	0.23
BDD	1.43	0.40	1.55	2.94	1.43 ± 0.35	1.44 ± 0.46	0.23	0.82	0.02

AA, attachment anxiety; ARS, appearance-based rejection sensitivity; BDD, body dysmorphic disorder.

The Pearson correlations are shown in **Table 3**. The results indicated that AA was significantly positive related to ARS (*r* = 0.41, *p* < 0.001) and BDD (*r* = 0.33, *p* < 0.001), while ARS was significantly positive related to BDD (*r* = 0.47, *p* < 0.001). Because relationship status was significantly correlated with attachment anxiety, it was included as a covariate in both the mediator-variable model and the dependent-variable model.

**Table 3:** Pearson correlations among variables.

Variable	1	2	3	4	5	6	7	8	9	10
1. Age	–									
2. Sex	-0.04	–								
3. Only child	0.21^***^	-0.05	–							
4. Romantic relationship	0.07^*^	-0.07	-0.04	–						
5. SES	-0.07	-0.16^***^	-0.24^***^	0.05	–					
6. Father education	-0.21^***^	-0.02	-0.32^***^	0.06	0.33^***^	–				
7. Mother education	-0.20^***^	-0.04	-0.33^***^	0.04	0.32^***^	0.80^***^	–			
8. AA	-0.03	-0.05	0.02	-0.12^***^	-0.08^*^	-0.05	-0.04	–		
9. ARS	0.01	-0.11^**^	0.02	-0.05	0.00	0.03	0.02	0.41^***^	–	
10. BDD	0.04	0.01	0.07^*^	0.03	-0.07	0.00	-0.02	0.33^***^	0.47^***^	–

*p <.05. **p <.01. ***p <.001. AA, attachment anxiety; ARS, appearance-based rejection sensitivity; BDD, body dysmorphic disorder; Sex, 0, male; 1, female; Only child, 1, yes; 2, no; Romantic relationship, 1, in a romantic relationship; 2, not in a romantic relationship; SES, Subjective Economic Status; 1, very low; 2, low; 3, medium; 4, high; 5, very high; Father/Mother education, 1, junior high and below; 2, senior high; 3, bachelor; 4, master; 5, doctor.

### 3.2 Testing the mediating role of ARS

As shown in **Table 4**, after controlling for age, whether an only child, whether in a romantic relationship, SES, father education level, and mother education level, we found a significant positive predicting effect from AA on BDD (β = 0.09, *SE* = 0.02, *p* < 0.001). A strong positive association was also found between AA and ARS (β = 2.79, *SE* = 0.22, *p* < 0.001). The correlation between ARS and BDD was also significant (β = 0.03, *SE* = 0.003, *p* < 0.001). The indirect effect of AA on BDD was significant [β = 0.086, bootstrapped 95% *CI* = (0.065, 0.111)]. These results indicated that the effect of AA on BDD was partially mediated by ARS.

**Table 4:** Mediation model and moderated mediation model (N = 815).

		Mediation model			Moderated mediation model		
Process	Predictors	β	SE	t	β	SE	t
1. Mediator variable model (ARS)	Constant	-3.766	2.714	-1.387	-3.766	2.714	-1.387
	Age	0.137	0.137	1.000	0.137	0.137	1.000
	Only child	0.258	0.359	0.720	0.258	0.359	0.720
	Romantic relationship	-0.036	0.390	-0.092	-0.036	0.390	-0.092
	SES	0.119	0.232	0.511	0.119	0.232	0.511
	Father education	0.288	0.250	1.152	0.288	0.250	1.152
	Mother education	-0.018	0.255	-0.072	-0.018	0.255	-0.072
	AA	2.790	0.215	12.970^***^	2.790	0.215	12.970^***^
2. Dependent variable model (BDD)	Constant	1.15	0.199	5.782	1.100	0.200	5.490
	Age	0.010	0.010	0.949	0.010	0.010	1.000
	Only child	0.044	0.026	1.664	0.050	0.026	1.912
	Romantic relationship	0.066	0.029	2.314^*^	0.068	0.029	2.367^*^
	SES	-0.026	0.017	-1.504	-0.020	0.017	-1.166
	Father education	0.021	0.018	1.141	0.018	0.018	1.002
	Mother education	-0.010	0.018	-0.546	-0.009	0.019	-0.468
	AA	0.086	0.017	4.962^***^	0.088	0.017	5.085^***^
	ARS	0.031	0.003	12.077^***^	0.028	0.003	8.882^***^
	Gender				0.057	0.025	2.237^*^
	ARS*Gender				0.010	0.005	1.982^*^
		R^2^ = 0.257, F = 34.872, df_1_ = 8, df_2_ = 806			R^2^ = 0.265, F = 28.972, df_1_ = 10, df_2_ = 804		
Process				Condition	Effect	Bootstrapped SE	Bootstrapped 95% CI
3. Conditional indirect effects of AA on BDD through ARS according to gender				Male	0.078	0.013	[0.055, 0.104]
				Female	0.105	0.017	[0.073, 0.141]

*p <.05. **p <.01. ***p <.001. AA, attachment anxiety; ARS, appearance-based rejection sensitivity; BDD, body dysmorphic disorder; SES, subjective economic status. All βs are unstandardized coefficients.

### 3.3 Testing the moderated mediating model

According to the moderated mediating model (see **Table 4**), the interaction of ARS and gender was positively related to BDD, and the effect size was significant. This result indicated that gender moderate the relationship between ARS and BDD. The bootstrapped (5000 times) test also showed that the mediating effect was moderated by gender (see **Table 4**). Specifically, the indirect effect of AA on BDD was stronger among females [0.105, 95% *CI* = (0.073, 0.141)] compared to males (0.078, 95% *CI* = [0.055, 0.104]), although significant for both genders.

**Figure 2:**
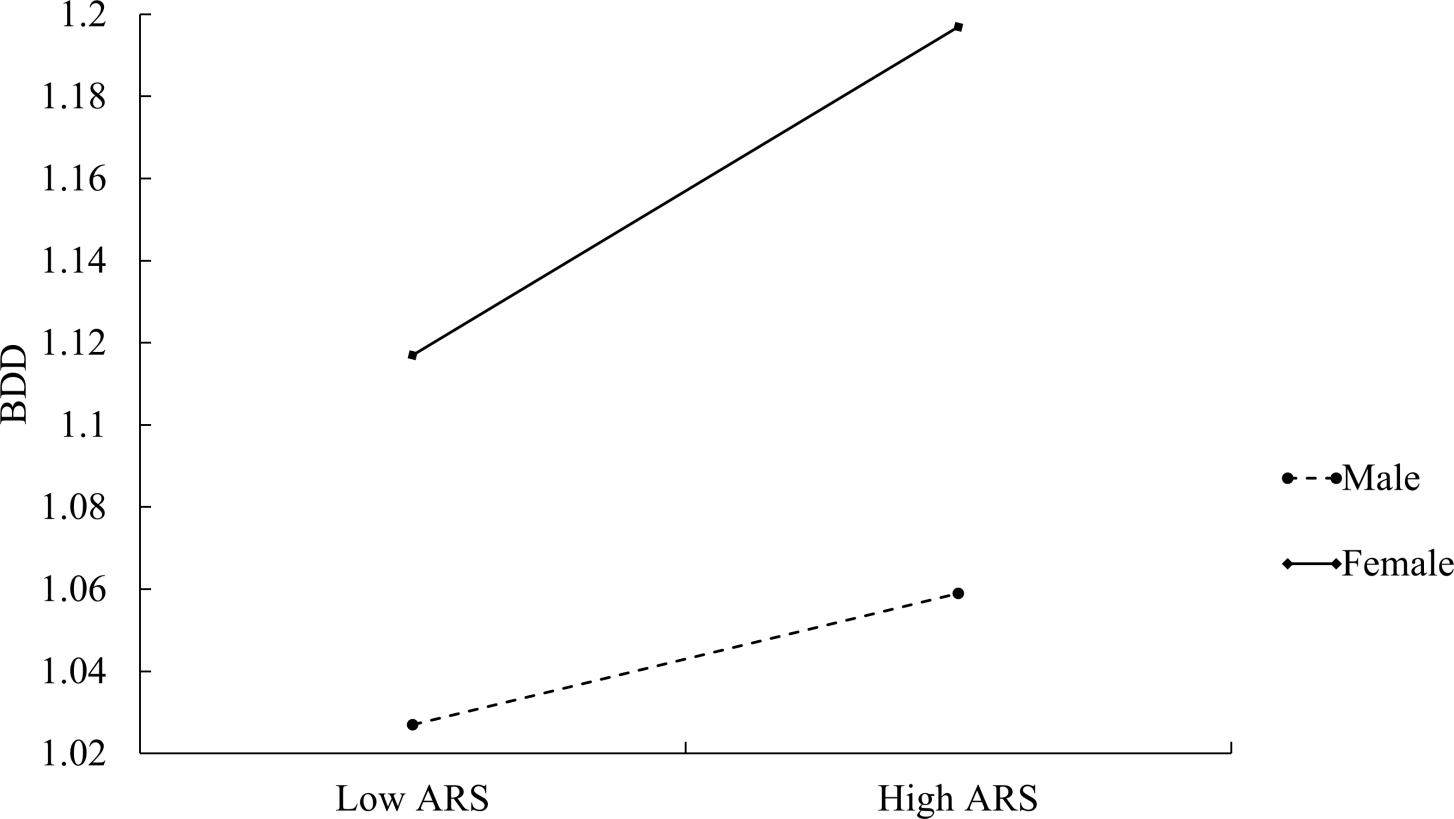
The relationship between ARS and BDD with gender as a moderator. ARS, appearance-based rejection sensitivity; BDD, body dysmorphic disorder.

The simple slope test was conducted better to understand the interaction effects of ARS and gender. As shown in **Figure 2**, the predicted effect of ARS on BDD among females (simple slope = 0.038, *SE* = 0.004, *t* = 9.403, *p* < 0.001) was stronger than males (simple slope = 0.028, *SE* = 0.003, *t* = 8.882, *p* < 0.001). Taking the above results into account, the Hypothesis 3 was supported.

## 4 Discussion

In our study, we analyzed data from 815 emerging young adults to establish a model linking attachment anxiety and BDD symptoms, with ARS serving as a mediator and gender as a moderator.

Our study established a direct relationship between attachment anxiety and BDD symptoms, highlighting the significant role of early relational experiences in shaping individuals’ perceptions of themselves (50). BDD, characterized by excessive concern over perceived flaws in appearance, often stems from an overvalued idea about body shape, leading to compulsive behaviors like frequent body checking. With previous research has explored various maladaptive cognitions associated with attachment anxiety, such as paranoia and attentional bias (51, 52), our study delves deeper into the developmental origins of BDD based on the results finding that inconsistent care during infancy can contribute to problematic real-world representations, which may manifest as BDD in early adulthood. By examining BDD through the lens of attachment theory, our study underscores the relational nature of this disorder, emphasizing the importance of early caregiving experiences in shaping individuals’ body image perceptions (53).

Recent scholarship has also established a notable correlation between attachment anxiety and the emergence of obsessive symptoms (54). Our empirical inquiries reinforced these assertions, spotlighting the pivotal role of attachment experiences in molding individuals’ self-concepts and relationship dynamics (55, 56). For young adults grappling with attachment anxiety, close interpersonal relationships serve as a secure base, while their self-identity often becomes undervalued (57, 58). Within this internal working model, individuals with attachment anxiety strive to attain close interpersonal interactions, sometimes resorting to excessive body checking as a means to alleviate perceived risks. Such behavioral patterns may exacerbate or manifest as symptoms of BDD, wherein individuals grapple with an excessive preoccupation over perceived flaws in their appearance to bolster self-esteem. Thus, comprehending the intricate interplay between attachment anxiety and BDD offers invaluable insights into the multifaceted dynamics underlying both conditions.

The current study not only identified the relationship between attachment anxiety and BDD but also delved into the underlying mechanism, pinpointing ARS as a pivotal bridge between the two. Previous research has consistently shown that individuals with attachment anxiety tend to possess a lower threshold for pain or rejection and may exhibit attentional biases towards negative stimuli, such as teasing (59–61). This heightened sensitivity stems from their strong desire for relationships, rendering them hypervigilant towards potential rejection from others (62). In the context of BDD, ARS, a specific dimension of rejection, may drive individuals to intensely scrutinize their appearance, seeking validation and reassurance.

Moreover, our study builds upon prior research on ARS and BDD (25, 27) by integrating the fundamental issue of attachment anxiety. By being hyperaware of potential negative feedback about their appearance, emerging adults may engage in defensive reactions, such as scrutinizing themselves for flaws to preemptively avoid perceived criticism or evaluation.

In contrast to previous research suggesting gender moderation in the association between ARS and BDD (28, 63, 64), our study revealed that females exhibited higher levels of BDD compared to males in our sample of Chinese emerging adults. We attribute this gender difference to societal norms surrounding body image, as documented by Etilé (65). In collectivist cultures like China, the importance of group identity and acceptance by peers is paramount (66). Moreover, females, in particular, often face stringent body standards in their daily lives and more pressure from peer, relatives, and social media (31). The pressure can lead to heightened sensitivity to rejection, especially concerning appearance (67, 68). This heightened sensitivity may contribute to the manifestation of BDD symptoms, such as obsessive body checking.

It is also noteworthy that, in the correlation analysis, being in a romantic relationship was negatively associated with attachment anxiety, suggesting that participants with higher attachment anxiety were less likely to report being currently partnered. Although this association was small, it is consistent with adult attachment theory and prior empirical findings. Individuals high in attachment anxiety often have a strong desire for closeness, yet they also hold negative self-models, fear rejection or abandonment, and remain highly vigilant to signs of relational threat (6, 13). These tendencies may complicate both the initiation and maintenance of romantic relationships. Previous research has shown that single young adults report higher attachment anxiety than partnered young adults and that worry about being rejected or unloved is a key attachment-related factor distinguishing single from partnered individuals (69). Meta-analytic evidence also indicates that attachment anxiety is associated with poorer romantic relationship quality and greater relationship conflict (70). Thus, the negative correlation observed in the present study may reflect the interpersonal difficulties and rejection concerns associated with attachment anxiety. In addition, the high proportion of partnered participants may have contributed to the relatively lower overall level of attachment anxiety in the sample.

At the same time, romantic relationship status was positively associated with BDD symptoms in the regression model after attachment anxiety, appearance-based rejection sensitivity, gender, and other covariates were controlled. BDD symptoms are often maintained by fears of negative evaluation, shame, reassurance seeking, appearance checking, and avoidance of situations in which perceived flaws may be noticed by others (15, 71). Romantic relationships involve intimacy, partner evaluation, attractiveness concerns, and the desire to maintain acceptance within a close relationship. In this context, perceived appearance defects may be experienced not only as a source of personal dissatisfaction, but also as potential threats to desirability, relational value, or partner acceptance. Prior research has shown that body image concerns are relevant to sexual and relational functioning in romantic relationships (72), and that partner objectification, romantic partner appearance commentary, attractiveness-contingent self-esteem, and romantic rejection concerns are associated with body dissatisfaction and appearance-related distress (73–75). Therefore, participants who were currently in romantic relationships may have been more likely to experience appearance concerns within an interpersonal context, which may partly explain the positive association between romantic relationship status and BDD symptoms observed in the regression model.

Females scored significantly higher than males on ARS in the present study. However, this gender difference should not be overstated. Although the difference was statistically significant, the effect size was small (Cohen’s *d* = 0.23). Prior research suggests that appearance-based rejection concerns are not limited to females. Webb et al. found that appearance-focused peer contexts, appearance conversations, appearance teasing, pressure to be attractive, internalization of appearance ideals, social comparison, and body dissatisfaction were associated with ARS among adolescents, and that these associations rarely differed between boys and girls (76). In subsequent research, they further discovered that appearance-related pressures from peers, parents, and media were associated with ARS, but that gender did not moderate these associations (77). Therefore, the present finding is best interpreted as evidence that females reported slightly higher ARS on average, rather than as evidence of a large or clinically meaningful gender difference.

## 5 Limitations

While our study contributes to understanding the fundamental mechanisms of BDD, it is essential to acknowledge several limitations for future research. First, the present sample consisted of a general college student population, rather than individuals with confirmed medical diagnoses. Further research is necessary to ascertain the generalizability of the current findings to clinical populations. Second, adopting a cross-sectional methodology, as common in BDD studies (18, 41), constrains our ability to establish causal relationships among attachment anxiety, ARS, and BDD. Employing longitudinal designs could address this limitation by tracing the development of BDD symptoms over time. Moreover, our sample predominantly comprised young adults, potentially limiting the generalizability of our findings across different age groups. Future studies should aim to include participants from diverse age cohorts to capture the developmental trajectory of BDD across the lifespan.

The sample composition should also be considered when interpreting the findings. Although the present study included both male and female participants, males were overrepresented, and most participants were currently in romantic relationships. These imbalances may limit the generalizability of the findings, particularly because body-image concerns and BDD-related symptoms may be expressed differently across gender groups, and because romantic relationship status was negatively correlated with attachment anxiety, suggesting that individuals with higher attachment anxiety who were not in romantic relationships may have been underrepresented. Moreover, given that gender moderated the association between ARS and BDD symptoms, sample composition may be relevant to the estimation of direct and indirect effects. Future studies should therefore recruit samples with more balanced distributions of gender and romantic relationship status, allowing the proposed model to be examined across male and female participants as well as single and partnered individuals.

## 6 Conclusions

This study investigated the association between attachment anxiety and BDD symptoms within a sample of early adults. We also explored the mediating effect of ARS and examined potential gender disparities in this relationship. The results revealed that attachment anxiety positively predicted BDD symptoms, with ARS mediating this relationship. Moreover, this mediation was more pronounced among females than males. These findings contribute both theoretical and empirical insights, offering groundwork for the advancement of more targeted prevention and intervention approaches for individuals suffered from BDD symptoms.

## Data Availability

The raw data supporting the conclusions of this article will be made available by the authors, without undue reservation.

## References

[1] American Psychiatric Association . Diagnostic and statistical manual of mental disorders, fifth edition, text revision (DSM-5-TR). Washington, DC: American Psychiatric Association Publishing (2022). doi: 10.1176/appi.books.9780890425787

[2] Nicewicz HR Torrico TJ Boutrouille JF . Body dysmorphic disorder. Treasure Island (FL: StatPearls (2024). 32310361

[3] Schneider SC Mond J Turner CM Hudson JL . Sex differences in the presentation of body dysmorphic disorder in a community sample of adolescents. J Clin Child Adolesc Psychol. (2019) 48:516–28. doi: 10.1080/15374416.2017.1321001 28541768

[4] Heshmati R Pellerone M Esfandi MRN Yeganeh N Jafari E . Interpersonal attachment styles and body dysmorphic symptoms in adolescent girls: the mediating role of body image. Clin Neuropsychiatry. (2023) 20:141–50. doi: 10.36131/cnfioritieditore20230206 37234357 PMC10206641

[5] Bowlby J . Attachment. New York: Basic Books (1969).

[6] Mikulincer M Shaver PR . An attachment perspective on psychopathology. World Psychiatry. (2012) 11:11–5. doi: 10.1016/j.wpsyc.2012.01.003 22294997 PMC3266769

[7] Mikulincer M Shaver PR Pereg D . Attachment theory and affect regulation: the dynamics, development, and cognitive consequences of attachment-related strategies. Motivat Emotion. (2003) 27:77–102. doi: 10.1023/A:1024515519160 41886696

[8] Mikulincer M Shaver PR . Attachment orientations and emotion regulation. Curr Opin Psychol. (2019) 25:6–10. doi: 10.1016/j.copsyc.2018.02.006 29494853

[9] Ainsworth MDS Blehar MC Waters E Wall S . Patterns of attachment: A psychological study of the strange situation. New York: Psychology Press (2014). doi: 10.4324/9781315802428

[10] PietroMonaco PR Barrett LF . The internal working models concept: What do we really know about the self in relation to others? Rev Gen Psychol. (2000) 4:155–75. doi: 10.1037/1089-2680.4.2.155 27371692

[11] Fraley RC . Attachment stability from infancy to adulthood: meta-analysis and dynamic modeling of developmental mechanisms. Pers Soc Psychol Rev. (2002) 6:123–51. doi: 10.1207/S15327957PSPR0602_03 42207527

[12] Pinquart M Feußner C Ahnert L . Meta-analytic evidence for stability in attachments from infancy to early adulthood. Attach Hum Dev. (2013) 15:189–218. doi: 10.1080/14616734.2013.746257 23210665

[13] Collins NL Read SJ . Adult attachment, working models, and relationship quality in dating couples. J Pers Soc Psychol. (1990) 58:644–63. doi: 10.1037/0022-3514.58.4.644 14570079

[14] Hazan C Shaver P . Romantic love conceptualized as an attachment process. J Pers Soc Psychol. (1987) 52:511–24. doi: 10.1037/0022-3514.52.3.511 3572722

[15] Fang A Wilhelm S . Clinical features, cognitive biases, and treatment of body dysmorphic disorder. Annu Rev Clin Psychol. (2015) 11:187–212. doi: 10.1146/annurev-clinpsy-032814-112849 25581240

[16] Fassnacht DB Ali K Kyrios M . Extending the cognitive-behavioral model of body dysmorphic disorder: the role of attachment anxiety and self-ambivalence. J Obsessive-Compuls Relat Disord. (2023) 37:100803. doi: 10.1016/j.jocrd.2023.100803 38826717

[17] Kelly MM Didie ER Phillips KA . Personal and appearance-based rejection sensitivity in body dysmorphic disorder. Body Img. (2014) 11:260–5. doi: 10.1016/j.bodyim.2014.03.004 24958661 PMC4519841

[18] Park LE . Appearance-based rejection sensitivity: implications for mental and physical health, affect, and motivation. Pers Soc Psychol Bull. (2007) 33:490–504. doi: 10.1177/0146167206296301 17363761

[19] Westen D . Towards a clinically and empirically sound theory of motivation. Int J Psychoanalys. (1997) 78:521–48. 9257166

[20] Toma CL . Online dating and psychological wellbeing: a social compensation perspective. Curr Opin Psychol. (2022) 46:101331. doi: 10.1016/j.copsyc.2022.101331 35349878

[21] De Paoli T Fuller-Tyszkiewicz M Krug I . Insecure attachment and maladaptive schema in disordered eating: the mediating role of rejection sensitivity. Clin Psychol Psychother. (2017) 24:1273–84. doi: 10.1002/cpp.2092 28488365

[22] De Paoli T Fuller-Tyszkiewicz M Halliwell E Puccio F Krug I . Social rank and rejection sensitivity as mediators of the relationship between insecure attachment and disordered eating. Eur Eat Disord Rev. (2017) 25:469–78. doi: 10.1002/erv.2537 28752904

[23] Bintaş-Zörer P Dirik G Department of Psychology, Faculty of Letters, Dokuz Eylul University, Izmir, Turkey . Social anxiety from an attachment theory perspective: the mediating role of early maladaptive schema domains and rejection sensitivity. JEBP. (2023) 23:25–47. doi: 10.24193/jebp.2023.2.9

[24] Set Z . Potential regulatory elements between attachment styles and psychopathology: rejection sensitivity and self-esteem. Arch Neuropsychiatry. (2019) 56:205–212. doi: 10.29399/npa.23451 31523148 PMC6732807

[25] Calogero RM Park LE Rahemtulla ZK Williams KCD . Predicting excessive body image concerns among British university students: the unique role of appearance-based rejection sensitivity. Body Img. (2010) 7:78–81. doi: 10.1016/j.bodyim.2009.09.005 19837638

[26] Farrell LJ Gregertsen EC Donovan CL Pammenter A Zimmer-Gembeck M . Maternal rejection and idealized value of appearance: exploring the origins of body dysmorphic concerns among young adults. J Cognit Psychother. (2016) 30:154–67. doi: 10.1891/0889-8391.30.3.154 32755921

[27] Densham K Webb HJ Zimmer-Gembeck MJ Nesdale D Downey G . Early adolescents’ body dysmorphic symptoms as compensatory responses to parental appearance messages and appearance-based rejection sensitivity. Body Img. (2017) 23:162–70. doi: 10.1016/j.bodyim.2017.09.005 29054091

[28] Webb HJ Zimmer-Gembeck MJ Mastro S Farrell LJ Waters AM Lavell CH . Young adolescents’ body dysmorphic symptoms: associations with same- and cross-sex peer teasing via appearance-based rejection sensitivity. J Abnorm Child Psychol. (2015) 43:1161–73. doi: 10.1007/s10802-014-9971-9 25582320

[29] Cooke JE Racine N Plamondon A Tough S Madigan S . Maternal adverse childhood experiences, attachment style, and mental health: pathways of transmission to child behavior problems. Child Abuse Negl. (2019) 93:27–37. doi: 10.1016/j.chiabu.2019.04.011 31048134

[30] Xiao S Asadullah MN . Social norms and gender differences in labor force participation in China. Feminist Econ. (2020) 26:114–48. doi: 10.1080/13545701.2020.1758337 37339054

[31] Boroughs MS Krawczyk R Thompson JK . Body dysmorphic disorder among diverse racial/ethnic and sexual orientation groups: prevalence estimates and associated factors. Sex Roles. (2010) 63:725–37. doi: 10.1007/s11199-010-9831-1 30311153

[32] Gonzales M Blashill AJ . Ethnic/racial and gender differences in body image disorders among a diverse sample of sexual minority U.S. adults. Body Img. (2021) 36:64–73. doi: 10.1016/j.bodyim.2020.10.007 33171428 PMC7987714

[33] Cook B Hausenblas H Rossi J . The moderating effect of gender on ideal-weight goals and exercise dependence symptoms. J Behav Addict. (2013) 2:50–5. doi: 10.1556/JBA.1.2012.010 26165771

[34] Keating C Stephens J Thomas N Castle DJ Rossell SL . Gender differences in weight‐related and non‐weight‐related appearance concerns in a community sample. Aust J Psychol. (2016) 68:11–9. doi: 10.1111/ajpy.12092 40046247

[35] Malcolm A Pikoos TD Castle DJ Rossell SL . An update on gender differences in major symptom phenomenology among adults with body dysmorphic disorder. Psychiatry Res. (2021) 295:113619. doi: 10.1016/j.psychres.2020.113619 33278744

[36] Collins NL . Revised adult attachment scale 1996. doi: 10.1037/t19162-000

[37] Wu W Zhang W Liu X . The reliability and validity of adult attachment scale (AAS-1996 revised edition): a report on its application in China. J Sichuan Univ (Med Sci). (2004) 35:536–8. doi: 10.3969/j.issn.1672-173X.2004.04.025 15291121

[38] Teixeira RCR Ferreira JHBP Howat-Rodrigues ABC . Collins and Read Revised Adult Attachment Scale (RAAS) validity evidences. Psico. (2019) 50:e29567–e29567. doi: 10.15448/1980-8623.2019.2.29567

[39] Deng Y Chen Y Zhao W Huang Y Liu X . Reliability and validity test of appearance-based rejection sensitivity scale in Chinese college students. Chin J Clin Psychol. (2018) 26:21–5. doi: 10.16128/j.cnki.1005-3611.2018.01.005

[40] Park LE Calogero RM Young AF Diraddo AM . Appearance-based rejection sensitivity predicts body dysmorphic disorder symptoms and cosmetic surgery acceptance. J Soc Clin Psychol. (2010) 29:489–509. doi: 10.1521/jscp.2010.29.5.489 37804533

[41] Lavell CH Zimmer-Gembeck MJ Farrell LJ Webb H . Victimization, social anxiety, and body dysmorphic concerns: appearance-based rejection sensitivity as a mediator. Body Img. (2014) 11:391–5. doi: 10.1016/j.bodyim.2014.06.008 25023480

[42] Lu L Chen T Chen J Lin W . A self rating scale of body image. Chin Ment Health J. (2000) 14:299–302.

[43] Zhang J Jiang L . Progress in the study of body dysmorphic disorder. Adv Psychol. (2024) 14:25–35. doi: 10.12677/ap.2024.146376

[44] Tang H Lv J Jiang Y Diao Q . A clinical study on medication combined with psychological counseling for vitiligo of the liver-qi stagnation type. J Pract Trad Chin Med. (2021) 37:951–2.

[45] Harman D . A single factor test of common method variance. J Psychol. (1967) 35:359–78.

[46] Hair J Black WC Babin BJ Anderson RE . Multivariate data analysis. Upper Saddle River, New Jersey: Pearson Educational International (2009).

[47] Byrne BM . Structural equation modeling with AMOS. New York: Routledge (2016).

[48] Rosseel Y . Lavaan: an R package for structural equation modeling. J Stat Softw. (2012) 48:1–36. doi: 10.18637/jss.v048.i02

[49] Muller D Judd CM Yzerbyt VY . When moderation is mediated and mediation is moderated. J Pers Soc Psychol. (2005) 89:852–63. doi: 10.1037/0022-3514.89.6.852 16393020

[50] Veale D . Over-valued ideas: a conceptual analysis. Behav Res Ther. (2002) 40:383–400. doi: 10.1016/S0005-7967(01)00016-X 12002896

[51] Bosmans G Koster EHW Vandevivere E Braet C De Raedt R . Young adolescent’s confidence in maternal support: attentional bias moderates the link between attachment-related expectations and behavioral problems. Cognit Ther Res. (2013) 37:829–39. doi: 10.1007/s10608-013-9526-3 30311153

[52] Martinez AP Agostini M Al-Suhibani A Bentall RP . Mistrust and negative self-esteem: two paths from attachment styles to paranoia. Psychol Psychother: Theory Res Pract. (2021) 94:391–406. doi: 10.1111/papt.12314 33314565 PMC8451824

[53] Gawęda Ł Pionke R Krężołek M Prochwicz K Kłosowska J Frydecka D . Self-disturbances, cognitive biases and insecure attachment as mechanisms of the relationship between traumatic life events and psychotic-like experiences in non-clinical adults – a path analysis. Psychiatry Res. (2018) 259:571–8. doi: 10.1016/j.psychres.2017.11.009 29195191

[54] Doron G Derby DS Szepsenwol O Talmor D . Flaws and all: exploring partner-focused obsessive-compulsive symptoms. J Obsessive-Compuls Relat Disord. (2012) 1:234–43. doi: 10.1016/j.jocrd.2012.05.004 38826717

[55] Ainsworth MDS Blehar MC Waters E Wall S . Patterns of attachment. New York, NY: Psychology Press (2014). doi: 10.4324/9781315802428

[56] Bowlby J . Attachment and loss. volume II. Separation, anxiety and anger. New York, NY: Basic Books. (1973). p. 429.

[57] Jayamaha SD Girme YU Overall NC . When attachment anxiety impedes support provision: the role of feeling unvalued and unappreciated. J Family Psychol. (2017) 31:181–91. doi: 10.1037/fam0000222 27359303

[58] von Mohr M Silva PC Vagnoni E Bracher A Bertoni T Serino A . My social comfort zone: attachment anxiety shapes peripersonal and interpersonal space. iScience. (2023) 26:105955. doi: 10.1016/j.isci.2023.105955 36718368 PMC9883291

[59] Besser A Priel B . Emotional responses to a romantic partner’s imaginary rejection: the roles of attachment anxiety, covert narcissism, and self‐evaluation. J Pers. (2009) 77:287–325. doi: 10.1111/j.1467-6494.2008.00546.x 19076997

[60] Meredith PJ Strong J Feeney JA . The relationship of adult attachment to emotion, catastrophizing, control, threshold and tolerance, in experimentally-induced pain. Pain. (2006) 120:44–52. doi: 10.1016/j.pain.2005.10.008 16359795

[61] Walsh JJ Balint MG Smolira Sj DR Fredericksen LK Madsen S . Predicting individual differences in mindfulness: the role of trait anxiety, attachment anxiety and attentional control. Pers Individ Dif. (2009) 46:94–9. doi: 10.1016/j.paid.2008.09.008 38826717

[62] Campbell L Marshall T . Anxious attachment and relationship processes: an interactionist perspective. J Pers. (2011) 79:1219–50. doi: 10.1111/j.1467-6494.2011.00723.x 21299557

[63] Webb HJ Zimmer‐Gembeck MJ Waters AM Farrell LJ Nesdale D Downey G . Pretty pressure” from peers, parents, and the media: a longitudinal study of appearance‐based rejection sensitivity. J Res Adolesc. (2017) 27:718–35. doi: 10.1111/jora.12310 29152860

[64] Zimmer‐Gembeck MJ Rudolph J Pariz J . A cascade of rejection and appearance preoccupation: adolescents’ body dysmorphic symptoms and appearance rejection sensitivity over 4 years. Br J Dev Psycho. (2022) 40:17–34. doi: 10.1111/bjdp.12377 33891314

[65] Etilé F . Social norms, ideal body weight and food attitudes. Health Econ. (2007) 16:945–66. doi: 10.1002/hec.1251 17605138

[66] Shin SK Ishman M Sanders GL . An empirical investigation of socio-cultural factors of information sharing in China. Inf Manage. (2007) 44:165–74. doi: 10.1016/j.im.2006.11.004 38826717

[67] Buote VM Wilson AE Strahan EJ Gazzola SB Papps F . Setting the bar: divergent sociocultural norms for women’s and men’s ideal appearance in real-world contexts. Body Img. (2011) 8:322–34. doi: 10.1016/j.bodyim.2011.06.002 21775228

[68] Chen FF . Sensitivity of goodness of fit indexes to lack of measurement invariance. Struct Equat Model: A Multidiscip J. (2007) 14:464–504. doi: 10.1080/10705510701301834 37339054

[69] Adamczyk K Bookwala J . Adult attachment and relationship status (single vs. partnered) in Polish young adults. Psihologijske Teme. (2013) 22:481–500.

[70] Li T Chan D-S . How anxious and avoidant attachment affect romantic relationship quality differently: a meta-analytic review. Eur J Soc Psychol. (2012) 42:406–19. doi: 10.1002/ejsp.1842 41531421

[71] Veale D . Advances in a cognitive behavioural model of body dysmorphic disorder. Body Img. (2004) 1:113–25. doi: 10.1016/S1740-1445(03)00009-3 18089145

[72] Quinn-Nilas C Benson L Milhausen RR Buchholz AC Goncalves M . The relationship between body image and domains of sexual functioning among heterosexual, emerging adult women. Sex Med. (2016) 4:e182–9. doi: 10.1016/j.esxm.2016.02.004 27036088 PMC5005305

[73] Zurbriggen EL Ramsey LR Jaworski BK . Self- and partner-objectification in romantic relationships: associations with media consumption and relationship satisfaction. Sex Roles. (2011) 64:449–62. doi: 10.1007/s11199-011-9933-4 21475650 PMC3062032

[74] Carriere LJ Kluck AS . Appearance commentary from romantic partners: evaluation of an adapted measure. Body Img. (2014) 11:137–45. doi: 10.1016/j.bodyim.2013.12.003 24440335

[75] Harrington AG Overall NC . Women’s attractiveness contingent self-esteem, romantic rejection, and body dissatisfaction. Body Img. (2021) 39:77–89. doi: 10.1016/j.bodyim.2021.06.004 34175783

[76] Webb HJ Zimmer-Gembeck MJ Donovan CL . The appearance culture between friends and adolescent appearance-based rejection sensitivity. J Adolesc. (2014) 37:347–58. doi: 10.1016/j.adolescence.2014.02.008 24793381

[77] Webb HJ Zimmer-Gembeck MJ Waters AM Farrell LJ Nesdale D Downey G . Pretty pressure” from peers, parents, and the media: a longitudinal study of appearance-based rejection sensitivity. J Res Adolesc. (2017) 27:718–35. doi: 10.1111/jora.12310 29152860

